# Association of Sleep Architecture and Physiology with Depressive Disorder and Antidepressants Treatment

**DOI:** 10.3390/ijms22031333

**Published:** 2021-01-29

**Authors:** Peter Hutka, Michaela Krivosova, Zuzana Muchova, Ingrid Tonhajzerova, Andrea Hamrakova, Zuzana Mlyncekova, Juraj Mokry, Igor Ondrejka

**Affiliations:** 1 Clinic of Psychiatry, Jessenius Faculty of Medicine in Martin, Comenius University in Bratislava, University Hospital Martin, 03659 Martin, Slovakia; hutka6@uniba.sk (P.H.); zuzanamuchova.sl@gmail.com (Z.M.); hamrakova4@uniba.sk (A.H.); mlyncekova3@uniba.sk (Z.M.); 2 Department of Pharmacology, Jessenius Faculty of Medicine in Martin, Comenius University in Bratislava, 03601 Martin, Slovakia; krivosova6@uniba.sk (M.K.); juraj.mokry@uniba.sk (J.M.); 3 Department of Physiology, Jessenius Faculty of Medicine in Martin, Comenius University in Bratislava, 03601 Martin, Slovakia; ingrid.tonhajzerova@uniba.sk

**Keywords:** depression, depressive disorder, sleep, polysomnography, antidepressants

## Abstract

Sleep problems are frequently associated with the principal diagnostic criteria for many mental disorders. Alterations in the sleep of depressive patients are of high clinical significance because continuous sleep problems raise the chance of relapse, recurrence, or suicide, as well as the need for augmenting medications. Most antidepressants have been proven to influence the sleep architecture. While some classes of antidepressants improve sleep, others may cause sleep impairment. The successful treatment of depressive disorder also requires an understanding of the effects of antidepressants on sleep. This article briefly reviews the physiology of sleep and the typical alterations in the sleep architecture in depressive patients and updates the different effects of the majority of antidepressants including novel drugs in clinical practice on sleep. The summary of the updated scientific findings of the relationship between depression and sleep disturbances could be clinically beneficial in choosing the best medication for depressive patients with concurrent sleep disorders.

## 1. Introduction

Depression, also known as depressive disorder, is a severe and common mood disorder that affects more than 264 million people worldwide. Recent systematic analysis showed that the global prevalence of depression increased by 33.4% from 1990 to 2007 and an increasing trend by 14.3% continued from 2007 to 2017 [1]. The progressive increase in incidence and prevalence of depressive disorder and the related suicide risk are considered as important reasons why depression has been studied by specialists for many years [1,2]. The etiology of depression is multifactorial, including impairment of neurotransmitters, synaptic plasticity mechanisms, low-grade inflammation, genetic and epigenetic factors, early environmental factors, and recent social stress [3,4]. Current pharmacological treatment still has several weak sides such as the lack of early-onset response, the mild response to the treatment, low remission rate to the first antidepressant trial, and side effects as reasons for treatment non-compliance [5].

Both the DSM-5 criteria as well as ICD-11 include sleep disorders such as disrupted or excessive sleep in the core symptoms that are present in the diagnosis of depression [6,7]. Approximately 60–90% of patients with major depression suffer from insomnia and report difficulties in initiating and maintaining sleep, as well as early morning awakening and remaining awake. Insomnia is characterized by problems with falling asleep or waking too early in the morning. Moreover, these difficulties are responsible for daytime impairment [8]. Insomnia is often so intensive that patients experience their depression as generally being a sleep disorder. Additionally, midnocturnal insomnia is considered the most common residual symptom of depressive disorder [9,10]. In subjects with bipolar disorder, insomnia occurs during a depressive episode in 60% of cases, while 20–30% of patients complain about increased daytime sleepiness and prolonged sleep [11,12]. Treating insomnia by cognitive behavioural therapy also reduced depression symptomatology [13]. In addition, depressive patients also report other sleep problems such as restlessness and nonrestorative sleep, although of normal duration [14].

This review compiles the physiology of sleep and typical alterations in sleep architecture associated with depression and treatment with the majority of clinically used antidepressants including novel drugs. The knowledge of scientific findings on this topic could contribute to better understanding sleep disturbances commonly seen in depression and could be clinically beneficial in choosing the right treatment option.

## 2. The Physiology of Sleep

Sleep is a physiological phenomenon alternating with a state of wakefulness and is characterized by decreased neural excitability to stimuli [15,16]. Carskadon and Dement [17] reported a behavioural point of view, “sleep is a reversible behavioural state of perceptual disengagement from and unresponsiveness to the environment”. Sleep belongs to the most substantial physiological processes performed by the human body. It is considered as a recovery phase that prepares the organism for the next episode of wakefulness [18]. Sleep supports appropriate maintenance of cognition, mood, and memory and is also necessary for appropriate functioning of the immune and endocrine systems [19]. In depressive disorder, the abnormal sleep architecture is associated with many serious health problems such as obesity, diabetes, hypertension, heart failure, and other cardiovascular diseases [20,21]. A recent epidemiological study stated a bidirectional relation between depression and duration of sleep. Short sleep makes depressive disorder onset and recurrence in males and those over 60 years more probable and depression increases the risk of shortened sleep [22]. Apart from gender differences, sleep also changes throughout life. In women, the major turning point is menopause, while in men, the subjective as well as objective quality of sleep decreases continuously [23]. In patients with depression, age and illness exert a synergistic worsening effect on sleep electroencephalography (EEG) [24].

The physiological sleep architecture consists of non-rapid eye movement sleep (NREM) and rapid eye movement sleep (REM) [17]. There are typically four–six cycles of NREM and REM sleep during the night. Almost 80% of total sleep time is represented by stages of NREM sleep. According to classification by the American Academy of Sleep Medicine [25], NREM sleep consists of three stages that differ by depth of sleep [24]. N1 demonstrates the shallowest one and N3 reflects the deepest stage. The physiological sleep architecture is defined by generalized slowing of EEG activity that reflects the progress from sleepiness to shallow sleep (N1), the presence of sleep spindles and K-complex waveforms that occur with the onset of deeper sleep (N2), and synchronized delta waves (N3). The periods of slow-wave sleep (SWS; synonymous to N3 and delta waves) occur principally in the first half of the night. Stages of REM sleep are physiologically noticeable in the second half of the night and they lengthen with each following cycle of sleep. Detailed assessment of the sleep architecture is available using polysomnography (PSG), as discussed below. Characteristic features of sleep stages are summarized in Table 1.

## 3. The Regulation of Sleep

Arousal and sleep are dynamic physiological processes regulated through a complex and only partially understood network of activation and suppression of neurologic pathways from the brainstem through the cerebral cortex. Within included cell populations are cholinergic, noradrenergic, serotonergic, dopaminergic, and histaminergic neurons located in the pedunculopontine and laterodorsal tegmental (PPT/LDT) nuclei, the locus coeruleus, the dorsal and median raphe nuclei, and the tuberomammillary nucleus. This arousal system is inhibited during sleep by sleep-active GABAergic and galaninergic neurons of the ventrolateral preoptic (VLPO) nucleus, and, reciprocally, the VLPO nucleus is down-regulated by the arousal system [26].

After the suppression of wakefulness by forebrain sleep neurons, the brain alternates between NREM and REM sleep. REM sleep is controlled by two neuromodulatory systems that show opposite firing rates. Monoaminergic neurons cease to fire during REM sleep and are called REM-off cells, while cholinergic neurons become highly active. Glutamatergic and GABAergic neurons also play a role in REM sleep generation [27].

## 4. Polysomnography—A Non-Invasive Tool for Sleep Evaluation

Polysomnography (PSG) is currently considered as a gold standard for investigating sleep disturbances. A graph received from PSG reflecting the sleep architecture is called a hypnogram. PSG consists of the registration of three main physiological parameters: EEG, which reflects the bioelectric activity of the brain, electromyography (EMG), which reflects the bioelectric activity of muscles, and electrooculography (EOG), which reflects eye movements. REM sleep is characterized by faster EEG activity, rapid and mostly horizontal eye movements viewed on EOG, instability in pulse and respiratory rates, and hypotonia of skeletal muscle seen on EMG [17]. Specifically, the PSG parameters characterize sleep continuity, depth, and distribution of sleep stages. It is important to also note the developmental aspect as several sleep parameters change during life. With increasing age in adults, absolute levels of SWS decrease, REM latency shortens, daytime sleep propensity reduces, and sleep latency and time awake during the night increase [28]. On the other hand, REM density does not show age dependence. REM density is also considered to be a reliable sleep marker for depression [29]. Short definitions of important sleep parameters and the relating age specificities are summarized in Table 2.

## 5. Sleep Disturbances Associated with Depressive Disorder

Depression is associated with several sleep architecture abnormalities that are recognized using PSG examination. Affected sleep parameters can be classified into three characteristic groups: parameters relating to sleep continuity, sleep depth, and distribution of sleep stages [30]. Following alterations in sleep parameters have been observed in depressive patients. First, disrupted sleep continuity manifests as prolongation of sleep latency. Concurrently, an increased number and duration of awakenings can occur as raised wake after sleep onset time, reduced sleep efficiency, and early morning awakenings. Early morning awakenings in connection with altered distribution of REM sleep are typical biological markers of depression with melancholic features [31]. Secondly, reduced sleep depth is a characteristic abnormality for patients with depressive disorder. Thirdly, the distribution of delta sleep varies in depressive patients, e.g., the highest delta waves activity of healthy individuals’ EEG manifests in the first sleep cycle. On the contrary, sleep parameters of depressive patients typically show a reduced delta ratio, and there is also a frequent shift in delta activity from the first to the second sleep cycle. REM sleep conversions are considered the most characteristic feature of the sleep architecture in patients with depression. More specifically, studies mention shortened REM sleep latency, increased REM sleep time in the first sleep cycle, and increased REM percentage, which is the amount of time an individual spends in the REM phase [10]. Sleep alterations in parameters such as REM latency probably have the potential to predict treatment response and the clinical progress of the disorder [30]. A decreased rapid eye movement onset latency (ROL) and delta sleep ratio can be explained by a cholinergic–aminergic imbalance [32]. Monoaminergic inhibitory influence on the PPT/LDT cholinergic cells is weakened and/or cholinergic drive in the pontine reticular formation (PRF) is strengthened in depressive patients, which results in greater propensity for REM sleep and increased intensity of REM sleep.

## 6. The Effects of Antidepressants on Sleep 

Antidepressants represent a wide group of drugs frequently used in clinical practice. The main indication for antidepressant medication is depressive disorder, but they can also be used for the treatment of other mental disorders or chronic pain [33].

Antidepressants vary in their effect on sleep: while some alleviate problems with sleep, others may cause sleep disturbances that influence the treatment compliance [34]. It is important to choose an appropriate pharmacological treatment for each patient individually noting their subjective complaints on sleep disturbances or objective worsening of sleep architecture found out by PSG. Alterations in the sleep of depressive patients are of high clinical significance because continuous sleep problems raise the chance of relapse, recurrence, or suicide, as well as the need for augmenting medications. For this reason, it is crucial to understand what the preferred pharmacological treatment is in a depressive patient with clinically relevant insomnia symptoms [10,30].

For clear understanding of human sleep–antidepressant interactions, it is important to be familiar with the physiological functioning of neurotransmitters in the central nervous system. The ability of antidepressants to influence the amount of monoamines in the synapse seems to be crucial.

An increased amount of serotonin is presumably responsible mainly for the effects on REM sleep [35]. Antidepressants that work as selective 5HT_1A_ agonists significantly suppress REM sleep [36]. However, tryptophan depletion, a mechanism that leads to a decrease in serotonin acutely in the human brain, has been shown to reverse REM suppression caused by antidepressants [37]. The inhibition of reuptake of neurotransmitters into the nerve ending, mainly serotonin and norepinephrine, is a typical mechanism of functioning of many antidepressants [38]. The increase in noradrenergic and dopaminergic neurotransmission or activation of serotonergic 5-HT_2_ receptors worsens the quality of sleep. This is typical for several antidepressants such as serotonin and norepinephrine reuptake inhibitors (SNRI), norepinephrine reuptake inhibitors (NARI), monoamine oxidase inhibitors (MAOI), selective serotonin reuptake inhibitors (SSRI), and activating tricyclic antidepressants (TCA) [10]. However, monoamine oxidase inhibitors increase the amount of monoamines by preventing breakdown by this enzyme. 

Alterations in NREM sleep, initiation, and maintenance of sleep seem to also be dependent on the action of other neurotransmitters [38]. Antidepressants with antihistaminergic action, such as sedating TCA, mianserin, or mirtazapine, act on the autoreceptors, thus supporting homeostatic maintenance of monoamine levels, blocking their negative feedback action, and therefore indirectly increasing monoamine levels. The sleep of some patients treated by mirtazapine already becomes better after the first drug dose [39]. Moreover, trazodone and nefazodone are antidepressants which, thanks to their strong antagonistic action at serotonergic 5-HT_2_ receptors, tend to promote sleep and improve its continuity [38]. 

The effects of antidepressants seem to be most significant and consistent on REM sleep. They appear to be the opposite to typical alterations for major depression [40]. Reductions in the amount of REM sleep and increases in REM sleep onset latency were found after taking antidepressant medications. Most antidepressants suppress REM sleep in both patients and healthy volunteers [41].

Antidepressants that increase the amount of serotonin in the synapse belong to the most effective inhibitors of REM sleep. The decrease in REM percentage is most significant early in treatment and progressively diminishes during long-term treatment. The exceptions are monoamine oxidase inhibitors which often tend to cause the absence of REM sleep for many months. The effects of antidepressants on sleep initiation and maintenance are much less consistent among drugs. However, the mechanism responsible for the different effects of antidepressants on sleep continuity is not yet known [38]. One of the potential explanations could be the antagonism of H_1_ histamine or cholinergic receptors [42] or the antagonism of postsynaptic 5HT_2C_ receptors occurring in mirtazapine which promotes sleep. However, another antagonist of 5HT_2A_ receptors, nefazodone, has no significant effect on slow-wave sleep [38,43,44]. 

While suppression of REM sleep has been examined widely, the delta sleep ratio (DSR) has also been studied in recent years. The DSR has a tendency to rise during treatment [45], and it has been assumed that the successful effect of antidepressants normalizes both slow-wave activity (SWA) and DSR [46]. The changes in SWA, rather than in REM sleep, may be related to the clinical impact of antidepressant medication. It appears that there is a strong correlation between DSR and REM latency [47]. However, nefazodone is an antidepressant that does not suppress REM sleep or ameliorate the delta sleep ratio [48]. Therefore, a change in the DSR does not seem to be a basic condition for clinical improvement. Alterations in slow-wave sleep appear to be a trait rather than state phenomena in depressive subjects [38].

A summary of subchronic and chronic effects of different antidepressants on sleep can be found in Table 3.

### 6.1. Tricyclic Antidepressants (TCA)

TCA are antidepressants whose effect on sleep has been widely studied, although their clinical use has become less significant. They can be divided into activating TCA (clomipramine, desipramine, imipramine, nortriptyline, protriptyline) that worsen insomnia, or sedating TCA (amitriptyline, doxepin, trimipramine) that improve sleep. It seems that their activating effect is mediated via the increase in noradrenergic and dopaminergic neurotransmission or activation of serotonergic 5-HT_2_ receptors. Generally, they act on the autoreceptors which supports homeostatic maintenance of monoamine levels, blocking their negative feedback action and therefore indirectly increasing monoamine levels. On the other hand, the sedating effect is related to their antihistaminergic action. DeMartinis and Winokur [49] noted that amitriptyline and doxepin shorten sleep onset latency, increase total sleep time, decrease awakenings after sleep onset, suppress REM sleep, improve sleep efficiency, and represent an effective treatment for insomnia. Low-dose doxepin treatment was the only recommended antidepressant medication for sleep maintenance insomnia by AASM clinical practice guidelines [50]. Trimipramine, which is only a weak monoamine reuptake inhibitor, is the only TCA that is not a strong REM suppressor. There is a study that even found a significant increase in REM sleep after one-month trimipramine treatment [51]. More studies demonstrated its decreasing effect on sleep onset latency and its increasing effect on sleep efficiency and sleep times in the short term in both healthy volunteers and depressive patients [51,52,53,54]. Ware et al. [54] reported in the double-blind placebo-controlled study that these effects of trimipramine occurred during the first week and were still present after 4 weeks on antidepressant treatment.

The stimulating TCA increase sleep latency and sleep awakenings and decrease total sleep time. According to Gursky and Krahn [55], clomipramine has the strongest ability to suppress REM sleep. Imipramine is another TCA with a very strong REM suppression effect. This effect is strongest after a few days of TCA treatment and then slightly decreases. After the discontinuation of most TCA, there is an REM sleep rebound which may be due to an increase in cholinergic function [38]. Clomipramine, desipramine, and imipramine increase waking during the first night, but this effect disappears after a few days [52,54,56,57,58,59]. It seems that TCA do not influence sleep continuity after a few days [38], but Mendlewicz et al. [60] reported a decrease in awakenings after 35–46 days of antidepressant treatment with amitriptyline. Some TCA increase N2 sleep [61,62].

### 6.2. Tetracyclic Antidepressants (TeCA)

Amoxapine, maprotiline, mianserin, and mirtazapine are antidepressants with a four-cyclic structure.

Amoxapine is classified either as a second-generation tricyclic antidepressant or as a TeCA, although four rings in the molecular structure are not fully fused together. It has the strongest 5HT_2C_ receptor binding affinity and, in a recent study, was found to be the most frequently associated with somnolence as a side effect [63]. In an experimental study of Maudhuit et al. [64], chronic treatment of amoxapine in rats led to REM suppression, inhibition of paradoxical sleep, and an increase in its latency, but no significant change in SWS and sleep continuity contrary to acute treatment (increased number of awakenings and decreased SWS). Loas [65] previously found increased SWS in both bad sleepers and healthy volunteers during the whole period of amoxapine treatment (3–4 weeks). Brebbia et al. [66] found reduced REM sleep in a reciprocal manner to delta sleep.

Studies related to effect of maprotiline on the sleep architecture are rare. Specifically, the drug was found to suppress REM sleep and increase stage 2 in healthy controls [67], and, like the other TeCA, it can be capable of enhancing SWS [68].

Mianserin and mirtazapine are characterized by combined serotonergic–noradrenergic action. They work as antagonists at 5HT_2_, α_2_, and H_1_ receptors. Both seem to have an improving effect on sleep. Mianserin (at 60 mg/day) was shown by Mendlewicz et al. [69] to improve sleep efficiency, reduce total time awake, and increase N2 sleep and ROL in depressive patients in both acute and chronic drug nights. Maeda et al. [70] also found a slight decrease in total REM time and an increase in ROL at the first, third, and seventh nights and an increase in N2 only during the first night in healthy men at a 20 mg/day dose of mianserin. Schittecatte et al. [71] reported that increased total sleep time, improved sleep efficiency, and decreased stage awake percentage were all significant after 5 weeks of mirtazapine treatment in depressive patients at a maximum dose of 45 mg per day on average. According to Schmid et al. [39], total sleep time as well as sleep efficiency increased, and time of awakenings decreased just after the second day of mirtazapine treatment and persisted until the end of the observation. Mirtazapine was also shown to modestly increase ROL and sleep efficiency and decrease sleep onset latency in depressive patients and may cause daytime somnolence which decreases over time [71,72,73].

### 6.3. Inhibitors of Monoaminoxidase (MAOI)

MAOI represent a group of antidepressants that were widely studied in terms of their effect on human sleep. Nowadays, they are no longer commonly used in clinical practice because of their undesirable adverse effects. Moreover, they tend to worsen the quality of sleep because of increased monoamines neurotransmission and activation of serotonergic 5-HT_2_ receptors. Generally, they enhance the amount of serotonin, norepinephrine, and dopamine by preventing breakdown by the enzyme.

Both reversible and irreversible MAOI are REM suppressors with the irreversible type being much stronger. An older study [74] found that it takes 14–40 days after the start of phenelzine administration to completely suppress REM sleep. Landolt et al. [75] showed complete REM sleep suppression in 6 of 11 depressive patients after 5 weeks of treatment with phenelzine at daily doses of 30–90 mg. Time not spent in REM sleep was mostly replaced by N2 sleep. The highest magnitude of REM suppression appeared after about 7 days and maximum rebound effects occurred after about 10 days after discontinuation of treatment. Phenelzine and tranylcypromine were found to disrupt sleep continuity by an increase in wake after sleep onset (WASO) which persisted from the early beginning until 5 weeks of treatment. Bloise and Gaillard [76] found that moclobemide, a short-acting, reversible MAOI with preferential action on monoamine oxidase (MAO) type A, increased N2 sleep and decreased the total duration of REM sleep but did not change the number of REM cycles during the night in healthy human subjects at 4 mg/kg of body weight. At a 6.5 mg/kg dose, there was a significant decrease in total sleep duration in the second and third nights and a decrease in REM sleep with partial return towards baseline in the third night. Monti et al. [77] found decreased total wake time, increased total sleep time, increased N2, and slightly increased SWS in depressive patients after short-term (1–3 nights), intermediate-term (14–16 nights), and long-term (26–28 nights) moclobemide treatment. Decreased REM sleep as a percentage of total sleep and increased REM sleep latency were only significant after short-term and intermediate-term treatment. Slight REM suppression was also observed in healthy men by Steiger et al. [78]. There were not any observed significant effects of MAOI on delta sleep [75,76,77,79].

### 6.4. Selective Serotonin Reuptake Inhibitors (SSRI) and Serotonin Norepinephrine Reuptake Inhibitors (SNRI)

Treatment of depressive disorder has changed over the years. Nowadays, the first-line pharmacotherapy for depression is represented by SSRI and SNRI.

It seems that SSRI could affect sleep by two mechanisms: the first mechanism is responsible for REM suppression, and it is based on a raised amount of serotonin and is probably mediated via the postsynaptic 5HT_1A_ receptors [35]. The second mechanism is related to the stimulation of postsynaptic 5HT_2_ receptors, has a sleep-disrupting effect, and thus is responsible for sleep fragmentation [38].

All of the SSRI reduce the overall amount of REM sleep and increase ROL. As Wilson and Argyropoulos [38] noted in their review, citalopram, fluvoxamine, paroxetine, and sertraline decrease total REM sleep and increase ROL in acute (1–2 nights) and chronic (> 21 nights) administration in both healthy volunteers and depressive patients. Similar but weaker effects are observed with fluoxetine. The increase in ROL is most prominent a few days after the start of treatment with fluoxetine. The effects on REM sleep become less evident after several weeks of treatment as studies after 8 weeks do not tend to show significant reductions in REM [73]. REM sleep rebound was shown 4–12 days after fluoxetine withdrawal [80] and 6 days after citalopram withdrawal [81]. Depressive patients have a lower delta sleep ratio than healthy individuals and an increase in this ratio after successful antidepressant treatment might be caused by the delay of the first REM period caused by SSRI. This is supported by the fact that nefazodone, which does not cause REM suppression, does not improve the delta sleep ratio [73].

Effects of SSRI on non-REM sleep consist of increasing light (stage) sleep, number of arousals from sleep, and time spent awake at night in both healthy subjects and depressive patients. However, the magnitude of these effects is more prominent in healthy subjects as depressive patients have already disrupted sleep at the baseline. These effects generally diminish in a few days after treatment initiation [73], except for fluoxetine which continues to decrease sleep efficiency and number of awakenings [44]. There were not any changes found in SWS between the baseline and after-treatment values [82].

The effect of SNRI on sleep seems to relate to the increase in noradrenergic neurotransmission and activation of serotonergic 5-HT_2_ receptors. The group of SNRI is represented mostly by venlafaxine and duloxetine. Luthringer et al. [83], in their double-blind placebo-controlled study, showed that venlafaxine has a similar effect to the classical antidepressants, i.e., it decreases sleep continuity, increases ROL, and decreases total REM duration in doses of 75–225 mg per day. There have been two studies conducted so far assessing the effect of duloxetine on sleep using polysomnography. Chalon et al. [84] showed that duloxetine increased ROL and decreased total REM sleep duration in healthy humans. It improved sleep continuity at an 80 mg/day dose and decreased it at a 120 mg/day dose. A second study also showed that after 7–13 days of treatment with duloxetine at a 60 mg single dose, there was a similar effect on REM sleep, but it also increased N3 sleep in depressive patients [85]. Treatment with another SNRI, milnacipran, did not show significant changes on sleep PSG parameters. No effect was observed on REM sleep, and only one study found increased ROL [86]. The study of Rahmani et al. [87] reported increased total awake time and decreased NREM sleep after 7 days of milnacipran treatment of depressive patients.

### 6.5. Dopamine Noradrenaline Reuptake Inhibitors (DNRI)

Bupropion, a member of the DNRI group, increases dopamine and norepinephrine levels via their reuptake inhibition. The mechanism of its antidepressant action, however, is not fully understood yet. Krystal et al. [88], in their review, summarized the effect of bupropion on the sleep architecture and concluded that bupropion did not have a consistent REM-suppressing effect and it did not alter the sleep architecture. Only in one study was an SWS increase noted [89], and another study mentioned a higher number of awakenings during sleep [90]. In an older study [91], a decreased ROL was observed; however, in a more recent study by Schramm et al. [92], an increased ROL was found as the only significantly changed polysomnography variable after acute treatment with bupropion.

### 6.6. Serotonin Antagonist and Reuptake Inhibitors (SARI)

Another group of antidepressants that has an important role in treatment of depression is represented by SARI. This group includes trazodone and nefazodone. Both of these antidepressants work as weak inhibitors of serotonin reuptake as well as the antagonists of postsynaptic 5-HT_2_ receptors and H_1_ and α_1_ adrenergic receptors. It seems that due to these mechanisms, trazodone and nefazodone tend to promote sleep and improve its continuity [38].

Mouret et al. [93], in their study of depressive patients treated with 400–600 mg, found increased total sleep time, total N2 sleep, slow-wave sleep, and sleep efficiency and decreased N1 sleep, sleep latency, and number of awakenings after 26–28 days of trazodone treatment. ROL was not increased after 2 days but increased at the end of the study. Other studies showed REM suppression and increases in SWS in healthy volunteers taking lower doses of trazodone [94]. Van Bemmel et al. [95] also found increased ROL but their results did not show a significant increase in TST and SWS duration. They also reported REM suppression at 300 mg of trazodone per day during 5 weeks in depressive subjects. When polysomnography was performed on healthy men, trazodone significantly increased SWS and decreased stages 1 and 2, and no REM suppression was observed [96].

The data about the effect of nefazodone on sleep are more conclusive. A clinical trial by Rush et al. [44] reported increased sleep efficiency and decreased sleep awakenings without REM suppression or increased ROL in depressive patients. In healthy subjects, nefazodone has no effect or even increases REM sleep in some studies [97,98,99].

### 6.7. Dopamine Reuptake Inhibitor (DARI)

Amineptine belongs to the group of dopamine reuptake inhibitors. After 14 days of treatment with this antidepressant agent at 200 mg/day, REM sleep was increased, ROL decreased, stage 3 of sleep decreased, and WASO increased [100,101].

### 6.8. Noradrenaline Reuptake Inhibitors (NARI)

Reboxetine and viloxazine significantly suppressed REM sleep and increased ROL both after acute and chronic administration in patients with dysthymia [102,103]. Number of awakenings and time awake after sleep onset were increased only after an acute dose of reboxetine, and in chronic treatment, these values were similar to the baseline conditions. SWS was significantly reduced only after acute reboxetine treatment compared to placebo; after a longer treatment period, SWS was slightly, but not significantly, increased [102]. Viloxazine decreased the amount of sleep spent in stage N3 and increased the number of awakenings. SWS and REM sleep were reduced after 3 weeks of viloxazine treatment [103,104].

Atomoxetine, used for ADHD, has one of the highest binding affinities to the norepinephrine transporter (NET). In a recent study, atomoxetine was the second antidepressant drug most related to the odds of somnolence [63]. In a study evaluating the effects of atomoxetine on sleep, the drug significantly increased latency to REM sleep and total percentage of this sleep stage was slightly decreased. Atomoxetine treatment also decreased the number of night-time awakenings but as this study included children suffering from ADHD, the baseline polysomnographic parameters might differ from those with depressive disorder.

### 6.9. Novel Antidepressants

Agomelatine is considered as a non-sedative antidepressant drug. It works as an agonist at melatonergic M_1_ and M_2_ receptors and as an antagonist at serotonergic 5HT_2C_ receptors. It also seems to have sleep-promoting properties without the risk of sedation. Agomelatine was reported to increase sleep efficiency, TST, and SWS and, simultaneously, to not affect REM sleep or the number of sleep cycles. It also preserves the physiological organization of sleep cycles [10,105,106,107].

Ketamine has been recently approved as a novel rapid-onset antidepressant, primarily used as a total intravenous anesthetic. It acts as a non-competitive glutamatergic N-methyl-D-aspartate-receptor (NMDA) antagonist with dose-dependent pharmacodynamic effects [108]. Only a single low dose of ketamine has been reported to have a significant effect on treatment-resistant depression (TRD) comparable to conventional antidepressants [109]. In addition to positive effects on mood, ketamine increases SWS, memory, and plasticity [110]. The study of Ahnaou concluded that after ketamine subcutaneous injections in rats, REM suppression, lengthened ROL, and longer deep sleep bouts were observed [108]. Duncan et al. [111] investigated that both acute and subchronic (day 1 and day 2) ketamine administration increased total sleep and decreased waking which was associated with the extended remission of TRD. Slow-wave sleep seems to be a significant marker of neuronal plasticity that was observed not only in ketamine but also in other novel depression therapies [110].

Tianeptine, previously classified as a serotonin reuptake enhancer (SRE), has been recently found to work as a full agonist at the µ opioid receptor [112,113]. There has been only one study that investigated the effects of tianeptine on the sleep EEG of depressive patients, but it studied the sleep architecture only after the first and sixth weeks of treatment and there was no PSG recording during baseline [114]. This study did not find any effects of this drug on any of the sleep parameters between the 7th and 42nd days of the treatment. Further research regarding the effects of tianeptine on sleep that would include baseline sleep recording is required.

Vilazodone has been classified as a serotonin partial agonist-reuptake inhibitor with a 5HT_1A_ receptor partial agonist profile [115]. There is not enough information about the effect of vilazodone on the sleep architecture yet. One study [116] investigated the effect of single-dose vilazodone treatment in healthy men and found a strong REM suppression effect as well as increased SWS sleep and wakefulness.

Vortioxetine, as a novel antidepressant, exerts the action of a selective inhibitor of serotonin reuptake and a direct modulator of several serotonergic receptors. It acts as a 5HT_3_, 5HT_7_, and 5HT_1D_ receptors antagonist, a partial agonist of 5HT_1B_ receptors, and a 5HT_1A_ receptors agonist [117]. Wilson et al. [118] firstly described the effects of vortioxetine at the doses of 20 and 40 mg per day after 3 days of vortioxetine treatment. They observed an increase in WASO and N1 sleep. The effects on REM sleep were similar to that of paroxetine, with increased ROL and REM suppression. The ROL decrease was in relation to the plasma levels of vortioxetine. However, this study evaluated only acute effects of vortioxetine on sleep—within three days of drug administration. Another retrospective study using questionnaires found that vortioxetine improved both subjective sleep quality and quantity [119]. In the adverse drug reaction study of Eugene [63], vortioxetine was least associated with somnolence among thirty selected antidepressants. More polysomnographic studies are needed to confirm the effect of chronic vortioxetine therapy on the sleep architecture.

**Scheme 1:**
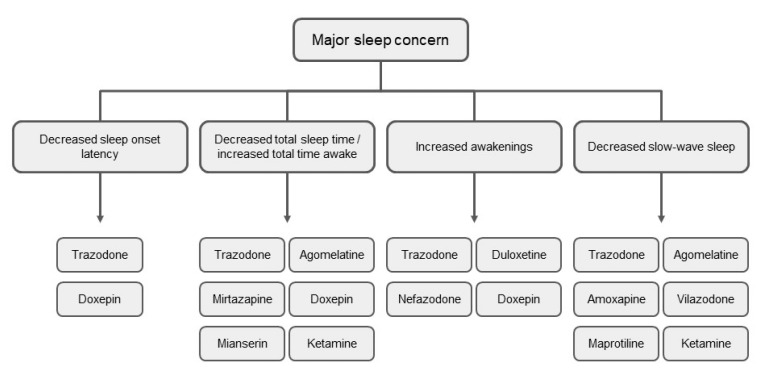
Antidepressants positively affecting sleep parameters.

Based on PSG studies, Scheme 1 summarizes and depicts antidepressants that have been shown to improve sleep problems related to insomnia.

## 7. Depression and Sleep in Children and Adolescents

Although several disrupted sleep patterns have been linked to depression in adults, alterations in PSG parameters in depressed children have been controversial. Studies show that results from depressed adult sleep polysomnography cannot be generalized to children and adolescents [120,121]. While some studies did not find any significant EEG differences between depressed and healthy children [122,123,124], others found similar sleep alterations in children with depressive symptoms to those in adults such as increased sleep latency, REM sleep, and number of awakenings and low sleep efficiency and proportion of SWS [125,126]. In young children (5–12 years), self-harm behaviour was associated with increased REM sleep [127]. Suicidal adolescents were found to have longer sleep onset latency, longer ROL, and a higher percentage of N1 sleep and REM density compared to matched healthy controls [128]. Other authors published that decreased baseline sleep efficiency and delayed sleep onset were associated with a later depression recurrence in children and adolescents [120] and even that longer sleep latency was a significant predictor of lifetime depression [129]. Fluoxetine use in depressive children and adolescents was associated with increased N1 sleep, number of arousals, and REM density but there was no change in ROL [130].

Further studies on the effects of depression and antidepressant treatments on the sleep architecture in children and adolescents are needed.

## 8. Conclusions

Sleep disturbances associated with depressive disorder represent a crucial problem for the selection of an antidepressant treatment. The successful treatment of depressive disorder requires understanding the effects of antidepressants on sleep. In this review, the effects of novel antidepressants such as agomelatine, ketamine, tianeptine, viloxazine, and vortioxetine on sleep, together with other more researched antidepressants, were discussed. Moreover, a scheme demonstrating antidepressants with positive effects on sleep parameters related to concomitant insomnia based on PSG studies was designed. In many cases, the exact mechanisms of antidepressants on sleep are still not fully understood. Thus, further research can elucidate novel knowledge linked to the pathomechanisms and effect of AD treatment on the sleep architecture, which is important for personalized therapeutic management of depressive patients, particularly those at the vulnerable adolescent age.

## Figures and Tables

**Table 1:** Characteristic features of sleep stages.

Sleep Stage	Characteristic Features
N1	❖ causing awakenings or arousals is easy ❖ the occurrence of slow eye movements ❖ brain wave activity begins to slow ❖ theta activity (4–8 Hz, 50–100 µV) is typical in EEG ❖ the presence of abrupt muscle spasms or hypnic jerks
N2	❖ causing awakenings or arousals becomes harder ❖ the slow eye movements discontinue ❖ brain waves continue to slow with specific sleep structures: sleep spindles and K-complexes ❖ body temperature decreases ❖ heart rate begins to slow
N3	❖ awakenings or arousals are rare ❖ the presence of delta waves (1–4 Hz, 100–200 µV) ❖ the most restorative stage of sleep ❖ the occurrence of parasomnias
REM	❖ awakenings and arousals can occur more easily ❖ brain waves are more active (15–30 Hz, < 50 µV) ❖ eye movements are rapid, moving from side to side ❖ the occurrence of hypotonia of skeletal muscle ❖ the presence of instability of pulse and respiratory rate

REM—rapid eye movement sleep, N1, N2, N3—non-rapid eye movement sleep stages, Hz—hertz, µV—microvolt.

**Table 2:** Definitions of sleep parameters and relating age specificities.

Parameter	Definition	Normal values	
		in Young Adult Patients	in Elderly Patients
**Parameters of sleep continuity**			
Sleep latency	The length of time that it takes to accomplish the transition from full wakefulness to sleep after the lights are turned off.	<30 min	<45 min
Total sleep time (TST)	The total time spent asleep during the sleep episode.	>6.5 h	>6 h
Sleep efficiency (SE)	Percentage of time spent asleep of the total time spent in bed.	>90%	>85%
Wake after sleep onset (WASO)	The length of time spent awake after the sleep onset.	<30 min	<30 min
**Parameters of sleep depth**			
Total and relative amounts of stage N3	Duration of N3 expressed as minutes or percentage relative to TST.	20–25%	10%
Delta sleep ratio (DSR)	The ratio of SWS in the first NREM period to SWS in the second.	>1.1	>1.1
**Parameters of REM sleep**			
REM latency	The length of time that it takes from onset of sleep to the start of the first REM episode.	<65 min	<50 min
Total and relative amounts of stage REM	Duration of REM expressed as minutes or percentage relative to TST.	20–25%	20–25%
REM density	Measure of the frequency of rapid eye movements during REM sleep expressed as movements per minute.	9–12 movements per min	9–12 movements per min

Modified according to Wichniak et al. [10].

**Table 3:** Summary of chronic effects of antidepressants on human sleep.

Antidepressant Class	Drugs	REM Sleep	ROL	SWS	Sleep Continuity
TCA (sedative)	amitriptyline, doxepin	↓	↑	↑	↑
	trimipramine	0/↑	0/↓	↑	↑
TCA (activating)	desipramine, imipramine, nortriptyline, protriptyline	↓	↑	↓	↓
	clomipramine	↓↓↓	↑↑↑	↓	↓
TeCA	amoxapine	↓	↑	0/↑	0
	maprotiline	0/↓	0	↑	0
	mianserin	↓	↑	↑	↑
	mirtazapine	0	0/↑	↑	↑
MAOI (reversible)	moclobemide	↓	↑	0/↑	↑
MAOI (irreversible)	phenelzine, tranylcypromine	↓↓↓	↑↑↑	0	0/↓
SSRI	citalopram, escitalopram, fluoxetine, fluvoxamine, paroxetine, sertraline	↓	↑	0	↓
SNRI	duloxetine, venlafaxine	↓	↑	↑	↓
	milnacipran	0	0/↑	0	0/↓
DNRI	bupropion	0	0	0/↑	0/↓
SARI	trazodone	0/↓	0/↑	↑	↑
	nefazodone	0/↑	0	↑	↑
DARI	amineptine	↑	0/↓	↓	↓
NARI	atomoxetine, reboxetine, viloxazine	↓	↑	0/↓	0/↓
Novel drugs	agomelatine	0	0	↑	↑
	ketamine	↓	↑	↑	↑
	tianeptine	0	0	0	0
	vilazodone	↓	↑	↑	↓
	vortioxetine	↓	↑	0	0/↓

Modified according to Wichniak et al. [10]; REM—rapid eye movement, ROL—rapid eye movement onset latency, SWS—slow-wave sleep, TCA—tricyclic antidepressants, TeCA—tetracyclic antidepressants, MAOI—monoamine oxidase inhibitors, SSRI—selective serotonin reuptake inhibitors, SNRI—serotonin norepinephrine reuptake inhibitors, DNRI—dopamine noradrenaline reuptake inhibitors, SARI—serotonin antagonist and reuptake inhibitors, DARI—dopamine reuptake inhibitor, NARI—noradrenaline reuptake inhibitors, ↑—increase, ↓—decrease, 0—no or minimal effect.
